# Utilizing Amniotic Fluid Metabolomics to Monitor Fetal Well-Being: A Narrative Review of the Literature

**DOI:** 10.7759/cureus.36986

**Published:** 2023-03-31

**Authors:** Charalampos Kolvatzis, Ioannis Tsakiridis, Ioannis A Kalogiannidis, Foteini Tsakoumaki, Charikleia Kyrkou, Themistoklis Dagklis, Angelos Daniilidis, Alexandra-Maria Michaelidou, Apostolos Athanasiadis

**Affiliations:** 1 Third Department of Obstetrics and Gynecology, School of Medicine, Faculty of Health Sciences, Aristotle University of Thessaloniki, Thessaloniki, GRC; 2 Department of Food Science and Technology, School of Agriculture, Faculty of Agriculture, Forestry and Natural Environment, Aristotle University of Thessaloniki, Thessaloniki, GRC; 3 Second Department of Obstetrics and Gynecology, Hippokration General Hospital, School of Medicine, Aristotle University of Thessaloniki, Thessaloniki, GRC

**Keywords:** nutrition, gestational diabetes mellitus, preterm delivery, metabolomics, fetal well-being, amniotic fluid

## Abstract

Fetal and perinatal periods are critical phases for long-term development. Early diagnosis of maternal complications is challenging due to the great complexity of these conditions. In recent years, amniotic fluid has risen in a prominent position in the latest efforts to describe and characterize prenatal development. Amniotic fluid may provide real-time information on fetal development and metabolism throughout pregnancy as substances from the placenta, fetal skin, lungs, gastric fluid, and urine are transferred between the mother and the fetus. Applying metabolomics to monitor fetal well-being, in such a context, could help in the understanding, diagnosis, and treatment of these conditions and is a promising area of research. This review shines a spotlight on recent amniotic fluid metabolomics studies and their methods as an interesting tool for the assessment of many conditions and the identification of biomarkers. Platforms in use, such as proton nuclear magnetic resonance (^1^H NMR) and ultra-high-performance liquid chromatography (UHPLC), have different merits, and a combinatorial approach could be valuable. Metabolomics may also be used in the quest for habitual diet-induced metabolic signals in amniotic fluid. Finally, analysis of amniotic fluid can provide information on exposure to exogenous substances by detecting the exact levels of metabolites carried to the fetus and associated metabolic effects.

## Introduction and background

In recent years, "omics" technologies have become popular and changed the scientific approach due to their role in describing diseases and biological systems through the mostly non-invasive analysis of large volumes of data [1,2]. Among these tools, metabolomics, offering new analytical techniques, explores and detects the set of low molecular weight molecules, including biological molecules, sugars, lipids, small peptides, vitamins, and amino acids, present in cells, tissues, organs, and biological fluids, mapping their phenotype into metabolic profiles [1,3]. The main advantages are high diagnostic power, high speed, convenience, and relatively low cost [1,4]. In contrast, traditional laboratory methodologies offer disease markers that often show low sensitivity or delayed onset [1,4].

Thorough metabolite analysis in biological fluids and different tissues can provide a comprehensive functional phenotype that incorporates clinical features and genetic and non-genetic factors [5], allowing evaluation of many metabolic responses to specific pathophysiological stimuli, such as drugs, environmental changes, lifestyle, diet, and other epigenetic factors [6,7]. Another advantage of metabolomics is the detection of rapid daily variability in the metabolic footprint, representing a metabolic "snapshot" [1,7]. Metabolic fingerprints provide information potentially related to the physiological states or pathological conditions of an organism; they can be produced by an array of analytical techniques, including proton nuclear magnetic resonance (^1^H-NMR) spectroscopy, gas chromatography-mass spectrometry (GC-MS), and liquid chromatography-mass spectrometry (LC-MS) [1].

The end phenotype is determined by environmental influences such as dietary and lifestyle, age, and intake of drugs or presence of disease [8]. Metabolomics analyses are a window to what actually happened since metabolites are the final products of gene, RNA, and protein interactions. On the other hand, genomics represents the potential outcome, while proteomics is the phenotypical outcome [1]. Metabolomics is a promising tool for the early diagnosis of various fetal, perinatal, pediatric, and adult diseases; they may also help monitor disease progression, optimizing treatment and evaluating relevant side effects in the perspective of personalized medicine [1,9-18].

Fetal and perinatal periods are critical phases of neonatal development. The stimuli and conditions to which the fetus is exposed are important factors for neonatal development [1]. Early diagnosis of various maternal complications is challenging due to their great complexity [15]. In addition, the central role played by the placenta during fetal life is highlighted by recent evidence; this structure represents a metabolic interface between the mother and the fetus, influencing neonatal maturation and metabolism [19]. There is a plethora of options about metabolomic substrates for fetal monitoring, prenatally, perinatally, and postnatally; the mother can provide amniotic fluid, placental tissue, blood, urine, breast milk, hair, and vaginal secretions, and the fetus/neonate can also offer urine, blood, saliva, bronchoalveolar fluid, exhaled air concentrate, feces, and umbilical cord tissue [20]. Applying metabolomics for the monitoring of fetal well-being, in the context of fetal growth restriction (FGR), gestational diabetes mellitus (GDM), preeclampsia (PE), preterm delivery, fetal infections, and other factors, is a promising area of research that could offer great insights in the understanding, diagnosis, and treatment of these conditions [15].

The scope of this narrative review was to synthesize available data on recent amniotic fluid metabolomics studies as an evolving tool for the assessment of many conditions and the identification of diagnostic and predictive biomarkers.

## Review

Amniotic fluid in metabolomics-based studies

Amniotic fluid has risen in a prominent position among metabolomics substrates in the latest efforts to describe and characterize prenatal development, being one of the more useful biological fluids utilized, as it reflects the metabolic profile of the fetus [21]. The amniotic fluid composition of carbohydrates, lipids, phospholipids, urea, and proteins reflects fetal and maternal health and is naturally altered during pregnancy [22,23]. It can provide information on the maturation or degradation of the fetus, especially the status of the developing lungs and kidneys, as well as substances used and biochemical exchanges [21]. In addition, before 20 weeks of gestation, the fetal skin is not keratinized and allows the two-way transport of soluble molecules between the amniotic fluid and the fetus, amniotic sac, placenta, and umbilical cord [24]. The amniotic fluid also contains cells from the kidneys, heart, lungs, liver, and the fetal hematopoietic lineage [25]. In combination, maternal blood and amniotic fluid provide metabolomics’ matrices for identifying fetal malformations, gestational diabetes mellitus, macrosomia, chromosomal diseases, spina bifida, preeclampsia, preterm delivery, FGR, and even metabolic errors of the neonate [26,27] or effects of the exposure to the fetus [28,29]. Metabolomics studies utilizing amniotic fluid as a sample matrix are mainly based on nuclear magnetic resonance (NMR) spectroscopy, gas chromatography-mass spectrometry (GC-MS), and liquid chromatography coupled to tandem mass spectrometry (LC-MS; LC-MS/MS), which allows the detection of a wide range of low molecular weight compounds present in the fluid [23,26]. Table 1 presents several prominent metabolites and their identification methods discussed in this article.

**Table 1:** Prominent metabolites in amniotic fluid studies LC-MS: Liquid chromatography–mass spectrometry; UPLC-MS/MS: Ultra performance liquid chromatography–tandem mass spectrometer; Q-ToF: Quadrupole time-of-flight mass spectrometry; HILIC: Hydrophilic interaction liquid chromatography; ESI: Electrospray ionization; ^1^H-NMR: Proton nuclear magnetic resonance; GDM: Gestational diabetes mellitus; PUFAs: Polyunsaturated fatty acids.

Condition	Method	Prominent metabolites in amniotic fluid	Studies
Pregnancy progression	^1^H-NMR proton freq.: 600.58 MHz. Sample temp.: 300 K. 1D (CPMG) NMR spin echo pulse sequence water suppression for filtering.	Disturbed glucose, carnitine, and amino acid levels. Higher levels of creatinine, electrolyte, pyruvate, choline, N,N-dimethylglycine, and urocanic salt. The L/P ratio was decreased in amniotic fluid and increased in maternal blood.	Orczyk-Pawilowicz et al. [27]
Preterm delivery	Targeted HILIC, UHPLC-MS with detection on a triple quadrupole mass spectrometer operating in both positive and negative electrospray ionization modes.	Higher glutamic acid, lower glutamine, pyruvate, and inositol in preterm labor. Higher glucose and taurine. Lower trimethylamine-n-oxide, choline, acetylcarnitine, creatine, theobromine, and uric acid.	Virgiliou et al. [30]
	Untargeted LC-MS amniotic fluid metabolomic profiling was analyzed using both reverse-phase and HILIC chromatography on a binary LC system coupled to a quadrupole time-of-flight mass spectrometer with electrospray ionization operated both in positive and negative ionization.	No evidence of grouping based on early mid-trimester.	Hallingström et al. [32]
Prematurity and bronchopulmonary dysplasia	Untargeted UHPLC-MS Q-ToF Synapt G2 interfaced with a UPLC system. Chromatographic analysis: reverse-phase HSS T3 column at 40°C. MS analysis with an electrospray source in both positive and negative ionization modes.	Higher levels of amino acids and their derivatives, unsaturated hydroxy fatty acids, oxylipins (putative metabolite: 4-hydroxy nonenal alkyne), and fatty aldehydes in premature labor women.	Baraldi et al. [33]
Gestational diabetes (GDM)	GC/MS on fast-scanning single-quadrupole mass spectrometer and LC/MS with UPLC coupled with a Q Exactive high-resolution/accurate mass spectrometer, and a UHPLC with a C18 column (2.1 × 150 mm, 3.5 µm) using a reversed-phase gradient with solvent A, of 0.2 mM ammonium fluoride in water, to solvent B, of methanol, at 0.2 mL/min flow rate for PUFAs analysis.	Lower levels of several amino acids (glycine, glutamine, histidine, lysine, phenylalanine, tryptophan, arginine, and indolepropionate) in GDM, only in male fetuses in mothers with GDM. Higher levels of tyrosine, leucine, methionine, and their metabolites in female fetuses transfer in mothers with GDM.	O’Neill et al. [24]
Fetal malformations and chromosomal defects			
Congenital diaphragmatic hernia	^1^H-NMR (25°C) no spinning using a 400 MHz NMR spectrometer equipped with a 5 mm [1H, 13C] inverse-detection dual-frequency probe	Leucine, valine, histidine, glutamic acid, electrolyte, lysine, glucose, creatinine acetate, lactate, α-oxoisovalerate, β-hydroxybutyrate, and β-aminobutyric acid showed altered levels.	Croitor-Sava et al. [42]
Down’s syndrome	LC-QTOF-MS. UPLC system – degasser, two binary pumps, and a thermostated autosampler (4°C) coupled with a Triple TOF 5600 mass spectrometer. With ESI for positive ion and negative ion modes. HILIC column, solvent A: water and 25 mM ammonium acetate and 25 mM ammonia, solvent B: acetonitrile.	30 metabolic pathways with variations in amino acid biosynthesis, ABC transporters, alanine, aspartate and glutamate, bile, neuroactive ligand-receptor interaction, galactose, arginine and proline, histidine, taurine, and hypotaurine. Higher levels of hydrocortisone and L-glutamine. Lower levels of coproporphyrin-III, L-glutamate, pregnenolone sulfate, taurochenodeoxycholic acid, L-arginine and taurocholic acid, L-histidine, and glycocholic acid.	Huang et al. [25]
Fetal sheep model of myelomeningocele	^1^H-NMR Rat 500 MHz with a temperature of 298 K	Glucose levels and oligosaccharides are higher in AF samples collected before reparative intervention. Increased Lac/Glc ratio after repair. Levels of my-inositol, lactic acid, alanine, isoleucine/leucine, and valine levels are considerably higher in AF samples after repair.	Ceccarelli et al. [43]
Chorioamnionitis	LC-MS/MS ESI+ mode. Capillary voltage 3.2 kV, source temperature 150°C, desolvation temperature 395°C, and dwell time 5 ms. Chromatographic separations were carried out using a C8 column (100 × 2.1 mm, 1.7 µm) running a gradient employing H_2_O (0.1% v/v formic acid) and acetonitrile (0.1% v/v formic acid) as mobile phases A and B.	Higher levels of inflammation markers, glutathione, 3-chloro-tyrosine, oxidative stress biomarker, and 8-hydroxy-2'-deoxyguanosine (8OHdG).	Cháfer-Pericás et al. [44]
	LC-MS/MS	IL4, IL10, IL12, and IL8 can be used as markers of mycosis.	Revello et al. [45]
Inflammation	(1) reverse-phase (RP) UPLC-MS/MS with positive ion conditions for hydrophilic compounds; (2) hydrophobic compounds; (3) negative ion conditions, and (4) HILIC/UPLC-MS/MS with negative ion conditions.	Higher levels of amino acids and purine.	Brown et al. [46]
Nutrition	^1^H-NMR (holistic approach) proton freq. 600 MHz triple resonance probe (HCN) at 25°C, CPMG pulse sequence to suppress protein signals of the untreated samples.	Higher levels of branched chain and aromatic amino acids in overweight women compared to normal women.	Athanasiadis et al. [47]
	^1^H-NMR (holistic approach) proton freq. 600 MHz triple resonance probe (HCN) at 25°C, CPMG pulse sequence to suppress protein signals of the untreated samples.	Higher concentrations of glucose, alanine, tyrosine, valine, citrate, cis-acotinate, and formate in the group of women were characterized by increased energy contributions from total and animal protein, saturated fatty acids, and elevated dietary glycemic index due to bad diet habits.	Fotiou [48]
	^1^H-NMR proton freq. 600 MHz triple resonance probe (HCN) at 25°C.	Higher levels of glucose, phenylalanine, histidine, valine, and alanine in the highest p/np quantiles and increased levels of lactic acid and choline in the lowest p/np quantiles.	Athanasiadou et al. [49]
	^1^H-NMR proton freq. 600 MHz triple resonance probe (HCN) at 25°C.	Higher levels of glucose, phenylalanine, histidine, valine, and alanine in the highest protein quantiles and increased levels of lactic acid and choline in the lowest protein quantiles.	Fotiou et al. [50]
Exposome effects on the fetus			
Maternal smoking	LC-MS coupled with dual liquid chromatography, alternating data collection between HILIC and C18 columns. Positive electrospray ionization mode. Injection volume of 10 μL, mass-to-charge ratio (m/z) scan range of 85 to 2000, and resolution of 60,000 (FWHM).	Higher levels of thymidine; proline and cytosine levels decreased; cytidine, triphosphate, and arginine levels increased. Conitine correlated with deregulation of fetal aspartic acid metabolism and the metabolism of many nucleic acids.	Fischer et al. [51]
Maternal pharmaceutical intake	LC-MS/MS C18 column (50 × 2.1 mm i.d.), particle size 2.6 μm; fitted with a 2.6-μm security guard cartridge (4 × 2.1 mm i.d.,). MS/MS triple quadrupole equipped with ES interface.	13 different phenethylamine derivatives.	Burrai et al. [52]
Twins complications			
Twin-twin transfusion syndrome	UHPLC-MS Dionex U3000 coupled to an electrospray LTQ-FT-MS ultra mass spectrometer and in both negative and positive ESI modes	Altered levels of acylcarnitines, acylglycerides, amino acids, carbohydrates, lipid metabolism, cholesterol esters, ceramides, sphingolipids, glycerophospholipids, nucleosides, oxidized lipids, and oxidative phosphorylation.	Dunn et al. [53]

Pregnancy progression

The metabolic pathways characteristic of amniotic fluid in normal pregnancies were summarized by Orczyk-Pawilowicz et al. via proton nuclear magnetic resonance spectroscopy (^1^H-NMR) [27] in the first study for normal pregnancies. A proton frequency of 600.58 MHz was utilized in the spectrometer, with a sample temperature of 300 K, and 1D Carr-Purcell-Meiboom-Gill (CPMG) NMR spin echo pulse sequence with water suppression was used for filtering out broad spectral resonances [27]. In particular, a metabolic change in 34 metabolites was observed, which was associated with the second- to third-trimester transition, followed by a pause, perhaps due to the stabilization of fetal growth [27]. There was a disturbance of glucose, carnitine, amino acids (valine, leucine, isoleucine, alanine, methionine, tyrosine, and phenylalanine) as well as higher levels of creatinine, electrolyte, pyruvate, choline, N,N-dimethylglycine, and urocanic salt. In addition, the lactate/pyruvate (L/P) ratio was observed to decrease in amniotic fluid and increase in maternal blood; this could be related to progressive changes in oxygen use by the mother and the fetus [27]. Since renal maturation profoundly affects the amniotic fluid composition, creatinine showed a large increase during the third trimester, followed by stabilization, possibly reflecting full renal maturation, a transition to a phase of rapid weight gain requiring anabolic processes [27]. Therefore, the reduction of amino acids required for protein synthesis and rapid growth, which plays a central role in energy balance and the tricarboxylic acid (TCA) cycle, has been shown to affect the amniotic fluid. In line with this, it is known that maternal metabolism exhibits anabolic characteristics in the first two trimesters of pregnancy, which then becomes predominantly catabolic in the third trimester, in contrast to what occurs in the fetus; pregnant women have to cope with the demands of increasing fetal metabolites, such as glucose, confirmed by the occurrence of maternal hypoglycemic episodes [27].

Preterm delivery

One out of 10 deliveries is preterm and can be associated with varying degrees of severity and highly variable outcomes [30]. The exact etiology has not been fully determined, but, possibly, it is multifactorial in nature. In a recent study by Virgiliou et al. [30], amniotic fluid samples during the second trimester of pregnancy were analyzed using ultra-high-performance liquid chromatography coupled with mass spectrometry (UHPLC-MS) to detect the possible predictors of preterm delivery or pregnancy outcome; the early metabolic profile of amniotic fluid was compared with controls, showing different groupings [30]. Among the 65 metabolites detected, glutamic acid was higher, while glutamine, pyruvate, and inositol were lower in preterm delivery together with several amino acids, probably due to an increased protein metabolism to sustain the increased fetal growth. Metabolites related to energy exchange were the most perturbed due to the higher-than-normal energy demands occurring in preterm delivery compared to early pregnancy [30]. Glucose and taurine were elevated in the amniotic fluid of preterm women, while trimethylamine-n-oxide, choline, acetylcarnitine, creatine, theobromine, and uric acid showed lower levels. The conclusion was that the fetal metabolic profile is dependent on maternal diet and gestational age, thus metabolomics could aid other methods and strategies focusing on nutrition to safeguard healthy fetal growth [30]. The method used in this study [30] was first developed on a HILIC system comprised of UHPLC with detection on a triple quadrupole mass spectrometer operating in both positive and negative electrospray ionization modes and validated with amniotic fluid samples from 250 second-trimester pregnant women [31]. On the contrary, based on mid-trimester metabolic profiling, there was no evidence of grouping about spontaneous preterm delivery according to a very recent study using untargeted LC-MS amniotic fluid metabolomic profiling [32]. Perhaps, extended targeted analysis on multiple pregnancy time points could offer clarification, but it is rather challenging due to the invasive sample collection method of amniocentesis.

Prematurity and bronchopulmonary dysplasia

Previous untargeted metabolomics data on 32 preterm infants aimed to predict preterm delivery, especially in relation to infectious/inflammatory intra-amniotic tracts [33]. Untargeted UHPLC-MS methods for amniotic fluid have been utilized to evaluate the untargeted methods for preterm delivery research and have found it promising with elevated levels of amino acids and their derivatives [33]. Prematurity is highly associated with both short- and long-term complications, with bronchopulmonary dysplasia being the most common and severe chronic disease [34]. Intra-amniotic inflammation and infections as well as placental dysfunction could be related to both preterm delivery and lung immaturity [21,33]. Metabolomics could detect preterm neonates who will later develop bronchopulmonary dysplasia, potentially paving the way for different targeted therapies. Several immunoassay studies have demonstrated the involvement of proinflammatory cytokines in bronchopulmonary dysplasia, the levels of which were increased in the amniotic fluid of women who subsequently delivered preterm [34-38]. Recent evidence has highlighted the combined predictive capacity of cytokines present in the amniotic fluid when used together with other metabolites [39], especially when utilizing artificial intelligence methods such as deep learning [40], while not utilizing metabolomics techniques offers new possibilities for investigation.

Gestational diabetes mellitus

GDM has adverse effects on fetal, neonatal, and infant metabolic outcomes by affecting glucose metabolism and insulin balance. GDM impairs the fetal-uterine balance and predisposes to glucose intolerance, obesity, and type 2 diabetes [21,41]. Therefore, metabolomics analysis of the amniotic fluid of pregnant women with GDM would be very useful [21]. Moreover, metabolomics could be useful for the early identification of women at high-risk for GDM; O'Neill et al. [24] found that 69 metabolites perturbed in GDM with the use of GC/MS on a fast-scanning single-quadrupole mass spectrometer, LC/MS with UPLC coupled with a Q Exactive high-resolution/accurate mass spectrometer, and a UHPLC with a C18 column using a reversed-phase gradient with solvent A, of 0.2 mM ammonium fluoride in water, to solvent B, of methanol, at 0.2 mL/min flow rate for polyunsaturated fatty acids (PUFAs) analysis. A large number of metabolites were perturbed in female-only fetuses (41 were increased), and 58 were perturbed exclusively in male-only fetuses (37 were decreased). In particular, a range of amino acids including glycine, glutamine, histidine, lysine, phenylalanine, tryptophan, arginine, and indolepropionate was decreased in GDM, only in male fetuses. Additionally, tyrosine, leucine, methionine, and their metabolites were increased mostly in female fetuses. Sexual differentiation probably leads to the differential expression of the metabolites in the two sexes, indicating a greater risk for GDM in women carrying male fetuses. Lipid metabolism was also affected by sex-related differences, with lower levels of medium-chain fatty acids (MCFAs) detected in male fetuses, while an increase in PUFAs and long-chain fatty acids (LCFAs) was noted in female ones. Fetal glucose, amino acid, glutathione, fatty acid, sphingolipids, and bile acid metabolism were also affected; glucose was higher due to increased placental transfer from mothers with GDM [24].

Fetal malformations and chromosomal diseases

A few metabolomic studies have addressed fetal malformations or chromosomal diseases, although metabolomics could aid in their understanding and early detection [25]. In particular, congenital diaphragmatic hernia, a potentially fatal malformation affecting approximately one to two in every 5,000 pregnancies, can be surgically treated in utero but with ill-defined success rates [42]. It appears that amniotic fluid samples can be used to distinguish fetuses with congenital diaphragmatic hernia and to characterize useful biomarkers with NMR spectroscopy [42]. Croitor-Sava et al. [42] detected metabolic changes of amniotic fluid in congenital diaphragmatic hernia, analyzing the amniotic fluid samples of 81 healthy and 22 affected fetuses. Leucine, valine, histidine, glutamic acid, electrolyte, lysine, glucose, creatinine acetate, lactate, α-oxoisovalerate, β-hydroxybutyrate, and β-aminobutyric acid showed variations between the two groups, even though they were related partially to congenital diaphragmatic hernia and gestational age differences [42].

Additionally, Ceccarelli et al. studied the impact of fetal myelomeningocele on metabolic pathways in amniotic fluid in a sheep model using ^1^H-NMR analysis [43]. The recent study by Huang et al. used UHPLC-Q-TOF-MS to evaluate amniotic fluid samples from 25 fetuses with Down’s syndrome and compared them with the same number of controls in the second trimester of pregnancy [25]. They detected altered metabolic pathways in the metabolism of porphyrin, bile acid, hormones, and amino acids; coproporphyrin-III, L-glutamate, pregnenolone sulfate, taurochenodeoxycholic acid, L-arginine, taurocholic acid, L-histidine, and glycocholic acid were significantly decreased, whereas hydrocortisone and L-glutamine were increased. Decreased levels of coproporphyrin-III may be related to the abnormal erythropoiesis evident in Down’s syndrome, while perturbations in glutamine and glutamate levels may be due to impaired neural development [25]. Nearly 30 metabolic pathways showed variations, including amino acid biosynthesis, ABC transporters, alanine, aspartate and glutamate, bile, neuroactive ligand-receptor interaction, galactose, arginine and proline, histidine, taurine, and hypotaurine, mainly in amino acid metabolism, liver development, and growth hormone regulation [25]. Even if such a study does not unambiguously elucidate all the metabolic patterns of Down’s syndrome, it reveals the promising role of metabolomics in investigating the etiology of diseases associated with abnormal chromosomes, functional consequences, and potential clinical manifestations [21]. Further studies integrating pathophysiology, genomics, epigenomics, and proteomics are indeed required [25].

Chorioamnionitis

Chorioamnionitis, an infection of the chorion and amnion, occurs when pathogenic microorganisms progress from the vagina into the uterus and infect the amniotic fluid, membranes, placenta, umbilical cord, and uterus [44-54]. It may occur in up to 20% of pregnancies and is associated with high morbidity and mortality for both the mother and the fetus, while its diagnosis can take up to seven days [55]. Chorioamnionitis exposes the fetus to oxidative stress and inflammation, leading to short-term conditions such as mycosis, chorionic vasculitis, vertical sepsis, prematurity, and long-term developmental problems [56,57]. Lipidomics studies link inflammation and lower levels of anti-inflammatory mediators with clinical chorioamnionitis [21,57] and oxidative stress using liquid chromatography coupled with tandem mass spectrometry [44]. Inflammation markers, glutathione, 3-chloro-tyrosine, oxidative stress biomarker, and 8-hydroxy-2'-deoxyguanosine (8OHdG) were significantly increased [44] (Table 1). Furthermore, in pregnancies with chorioamnionitis, lower birthweight and neonatal glucose levels were observed [45]. Cytokines also seem to play a role in amniotic fluid metabolomics as several interleukins such as IL4, IL10, IL12, and IL8 can be used as differential markers of mycosis in women at risk of chorioamnionitis [45] probably for pathogen identification [58].

Neurodevelopment

Inflammation can impair fetal development, particularly with regard to neurodevelopment [21]. Rat model metabolomics linked inflammation with brain maturation; fetal sex-related differences were detected in key metabolites for neuronal development in fetal and neonatal rat brain samples, using an induced uterine inflammation model that found increased amino acids and purine in amniotic fluid, even though many metabolites returned to their initial levels after 48 hours [46].

Nutrition

Moreover, metabolomics offers unique opportunities in attempting to probe the potential effect of maternal nutritional status on amniotic fluid composition [59,60]. It should be emphasized that “maternal nutritional status” does not only refer to nutrient intake but also reflects other nutritional indices, such as body mass index (BMI) [48].

**Figure 1:**
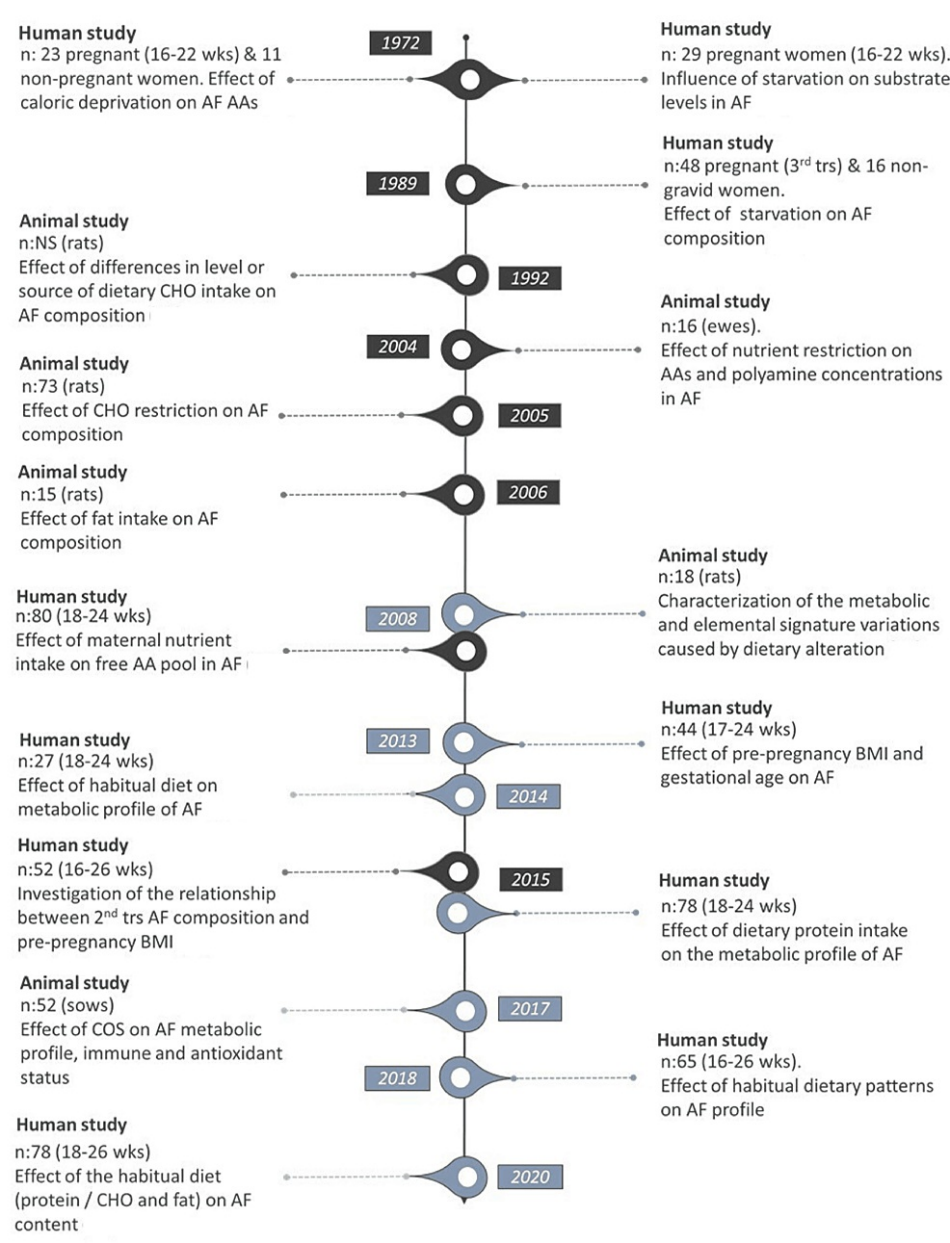
Overview of human and animal studies examining the potential effect of maternal diet on amniotic fluid composition Studies conducted by conventional analytical methods are presented in black color, while metabolomics studies are colored in gray. Extra timeline citations [65-74] are given in chronological sequence. AF: Amniotic fluid; BMI: Body mass index; CHO: Carbohydrates; COS: Chitosan oligosaccharide; NS: Non-specified; wks: Weeks; trs: Trimester; AA: Amino acids.

Over the past decades, there is sufficient evidence from animal and human research highlighting the critical role of maternal nutrition on amniotic fluid composition and consequently on the intrauterine milieu. In this direction, several approaches have been employed, while, in recent years, there is a shift toward adopting metabolomic approaches instead of conventional analytical protocols (Figure 1).

Athanasiadis et al. used a reversed-phase HPLC protocol based on precolumn derivatization of amino acids and ^1^H-NMR spectroscopy to explore the potential association between maternal BMI before pregnancy, gestational age (GA), and human amniotic fluid composition [47]. Data ensuing from the conventional analysis pointed toward distinguishing normal from overweight individuals; elevated levels of branched chain and aromatic amino acids were reported in overweight women. The ^1^H-NMR approach discriminated between the two BMI classes as well. The complementary application of the two methods indicated a compositional tendency in the metabolic profile of AF influenced by GA and maternal BMI before pregnancy [47].

In 2015, Fotiou et al., [50] to confirm previous research findings concerning the diet-induced changes in the free amniotic fluid amino acid pool [61], employed ^1^H-NMR for the identification of the metabolites related to habitual maternal diet expressed as protein intake. Seventy-eight pregnant women, at 18-24 weeks of gestation, were categorized based on protein intake (g/1000 kcal) into deciles, quartiles, and tertiles. Amniotic fluid samples of women belonging to the lowest quantiles were characterized by elevated levels of choline and lactate, while those retrieved from women in the upper quantiles presented increased concentrations of glucose and amino acids (valine, alanine, histidine, and phenylalanine) [50]. Additionally, maternal diet expressed as a ratio of protein to carbohydrate and fat (p/np) may also influence the amniotic fluid metabolite content (Table 1) as observed from the distinct separation of extreme groups in the orthogonal partial least squares discriminant analysis (OPLS-DA) scatterplots. Following the same statistical approach (deciles, quartiles, and tertiles) outlined above, Athanasiadou et al. concluded that the effect on amniotic fluid content was more evident when the comparisons were made between extreme groups with the greatest difference in p/np [49].

NMR-based metabolomics was also used to unravel the potential effect of maternal habitual dietary patterns on amniotic fluid profile [62,63]. In a recently published study, 65 women were categorized into two groups based on the consumption of 20 predefined food groups [63]. The authors reported that women characterized by increased energy contributions from total and animal protein, saturated fatty acids, and elevated dietary glycemic index exhibited higher concentrations in key metabolites (i.e., glucose, alanine, tyrosine, valine, citrate, cis-aconitate, and formate) in amniotic fluid compared to those characterized by higher consumption of plant protein, monounsaturated and polyunsaturated fatty acids [63]. The metabolic changes in amniotic fluid induced by maternal dietary habits could be associated with amino acid and glucose metabolism as well as the citric acid cycle. Further research is needed to elucidate whether the impact negatively affects the embryo in later life leading to obesity.

Exposome effects on the fetus

Environmental factors can be hostile to pregnancy, and they may affect the well-being of the fetus in a variety of ways. Maternal smoking as well as second-hand smoking, during pregnancy, can lead to FGR, prematurity, stillbirth, and sudden infant death syndrome [51]. Fischer et al. [51] used LC-MS to analyze second-trimester amniotic fluid samples from 81 pregnant women at low-level nicotine exposure. Conitine, a nicotine-derived metabolite, correlated with deregulation of fetal aspartic acid metabolism and the metabolism of many nucleic acids, asparagine, arginine, proline, pyrimidine, thymidine, proline, cytosine, cytidine, triphosphate, and arginine [51].

In addition, metabolomics can be used to detect traces of drug substances that have passed from mother to fetus; Burrai et al., in amniotic fluid samples from 194 pregnant women, assessed with LC-MS/MS and identified 13 different phenethylamine derivatives, a psychoactive group of controlled substances that include amphetamine, methamphetamine, and MDMA [52].

Twins’ complications

Monochorionic twin pregnancies are not without complications, including the twin-twin transfusion syndrome (TTTS) that can lead to cardiac overload and dysfunction in the twin recipient (70%), with a mortality rate of 90% if left untreated [64]. Fetal cardiac function can be monitored by analyzing the levels of cardiac troponin T and atrial natriuretic polypeptide in the amniotic fluid [53]. A recent study evaluated the amniotic fluid metabolomics of 19 women in twin pregnancies by UHPLC-MS, in relation to fetal cardiac function decline, before and after fetoscopic laser surgery. Acylcarnitines, acylglycerides, ceramides, sphingolipids, and glycerophospholipids as well as other fatty acids and oxidized lipids were negatively associated with cardiac function, while carbohydrates were positively associated. Hormones and two oxidative phosphorylation metabolites were also negatively associated with cardiac function, while changes in the concentration of N,N-dimethylarginine were also detected [53]. Following surgery, 200 metabolites changed significantly, with acylcarnitines, acylglycerides, amino acids, carbohydrates, lipid metabolism, cholesterol esters, ceramides, sphingolipids, glycerophospholipids, nucleosides, oxidized lipids, and oxidative phosphorylation being the most notable [53]. It seems that amniotic fluid metabolomics varies in relation to the severity of cardiac dysfunction and is also modified after relevant treatments [21]. Fatty acids seem to have a negative association with cardiac function, while carbohydrates show the opposite [53]. These results could help in monitoring cardiac function and could offer a reasonable degree of fetal prognosis for several twins complications [53].

Amniotic fluid metabolomics

The above-reviewed studies shine a spotlight on metabolomics as an evolving tool for the assessment of many conditions and as possible future biomarkers for improved therapeutic strategies. Different platforms are in use, such as ^1^H NMR and mass spectrometry in biological fluids. ^1^H NMR has lower sensitivity and resolution when compared to LC-MS, GC-MS, and HPLC, which have a large metabolite repertoire, but ^1^H NMR quantitative nature has advantages that still make it attractive to biological matrices’ metabolomics [75]. A combinatorial approach could be valuable but not always possible due to sample availability and conditions [76].

Studies carried out in recent years have provided interesting data on normal [27] and complicated pregnancies [30], such as metabolic changes in the second trimester, which seem to be associated with the transition to the third trimester, followed by a stabilization in fetal growth and neonatal outcome. In addition, predictive markers of prematurity appear to be applicable [33]. Furthermore, metabolites involved in the short- and long-term health of preterm neonates could be useful in predicting the outcome and complications that occur in these deliveries [42,44].

With regard to metabolic profile changes with nutrition, specific metabolic variations were seen in the amniotic fluid metabolism of mothers with GDM, which could underlie the different outcomes evident in the two sexes [48-50]. Metabolomics in fetal defect studies has been proven useful for the investigation of potential links between pathogenetics and clinical manifestation as well as to find prognostic markers of intra-amniotic infection [42,44]. Analysis of amniotic fluid provides actionable information on maternal nutrition or exposure to exogenous substances, detecting the exact levels of metabolites carried to the fetus and associated metabolic outcomes [51,52]. This innovative approach will lead to a lot of ethical issues regarding patient autonomy and the decision to treat fetuses as individual patients as intrauterine therapies expose mothers to well-documented but small risks with weighted increased benefits in serious cases [77-79]. These issues need to be earnestly addressed for the wide use of amniotic fluid testing.

## Conclusions

Finally, as a future target, metabolomics could prove useful in optimizing individualized treatment and nutritional guidance, assess drug-related efficacy or toxicity, identify phenotype changes associated with disease onset/progression and improve early diagnosis and prognosis. They could improve the accuracy of efficacy, paving the way for better clinical trials. Currently, a clear and well-defined association between each metabolite and its relative clinical importance is not available, although an atlas addressing the involvement of metabolites in many pediatric and neonatal diseases and conditions would be of value.
